# Recruitment for a clinical trial of chronic disease self-management for older adults with multimorbidity: a successful approach within general practice

**DOI:** 10.1186/1471-2296-14-125

**Published:** 2013-08-28

**Authors:** Richard L Reed, Christopher A Barton, Linda M Isherwood, Jodie M Oliver Baxter, Leigh Roeger

**Affiliations:** 1 Discipline of General Practice, Flinders Prevention Promotion and Primary Health Care Cluster, Flinders University, Health Sciences Building, Level 3, Registry Road, Bedford Park, South Australia, Australia; 2 Social Health Science, Flinders Prevention Promotion and Primary Health Care Cluster, Flinders University, Sturt Building, Bedford Park, South Australia, Australia; 3 National Institute of Labour Studies, Social and Behavioural Sciences, Flinders University, Social Sciences North Building, Bedford Park, South Australia, Australia

## Abstract

**Background:**

A robust research base is required in General Practice. The research output for General Practice is much less than those of other clinical disciplines. A major impediment to more research in this sector is difficulty with recruitment. Much of the research in this area focuses on barriers to effective recruitment and many projects have great difficulty with this process. This paper seeks to describe a systematic approach to recruitment for a randomized controlled trial that allowed the study team to recruit a substantial number of subjects from General Practice over a brief time period.

**Methods:**

A systematic approach to recruitment in this setting based on prior literature and the experience of the investigator team was incorporated into the design and implementation of the study. Five strategies were used to facilitate this process. These included designing the study to minimize the impact of the research on the day-to-day operations of the clinics, engagement of general practitioners in the research, making the research attractive to subjects, minimizing attrition and ensuring recruitment was a major focus of the management of the study.

Outcomes of the recruitment process were measured as the proportion of practices that agreed to participate, the proportion of potentially eligible subjects who consented to take part in the trial and the attrition rate of subjects. Qualitative interviews with a subset of successfully recruited participants were done to determine why they chose to participate in the study; data were analyzed using thematic analysis.

**Results:**

Five out of the six general practices contacted agreed to take part in the study. Thirty-eight per cent of the 1663 subjects who received a letter of invitation contacted the university study personnel regarding their interest in the project. Recruitment of the required number of eligible participants (n = 256) was accomplished in seven months. Thematic analysis of interviews with 30 participants regarding key factors in their study participation identified a personalised letter of endorsement from their general practitioner, expectation of personal benefit and altruism as important factors in their decision to participate.

**Conclusion:**

Recruitment can be successfully achieved in General Practice through design of the research project to facilitate recruitment, minimize the impact on general practice operations and ensure special care in enrolling and maintaining subjects in the project.

## Background

Many research questions most relevant to primary care can only be investigated in this setting yet much of the evidence used is generated in other clinical settings. Research findings extrapolated from hospital outpatient or inpatient settings often provide results that are misleading because a more severe spectrum of clinical conditions is seen in acute care. In addition determining the best way to organize and provide General Practice care can only be studied in this setting. Despite the centrality of a robust and evidence informed primary health care sector, research studies are much less likely to be performed in primary care than other sectors of health care resulting in important information gaps to inform policy and practice. This is particularly true when the rate of publications in this sector is expressed as the number of publications per practicing physicians in this sector. For example in Australia for the period 2000–2007, there were 3.0 publications produced per 1000 general practitioners per year which was less than 5% of the rate for surgeons during 2000–2007, and about 2% of that of internal medicine specialists [1].

One major barrier to research in General Practice is that recruitment of subjects in this setting is often very challenging [2,3]. Several reasons are commonly cited including lack of resources (time, staff and training) [4]; concerns about the effect on the professional–patient relationship [5]; insufficient interest, rewards or recognition [4]; [6]; and lack of a research ethos or culture in General Practice [7,8]. A range of solutions has previously been proposed to increase recruitment of subjects in this setting, including minimizing the complexity of the protocol [9,10], providing financial compensation to practices to pay for additional staff time required in participation in the study [11] building upon personal relationships with practice staff [12], use of electronic records to identify potential subjects [13] involvement of a discipline champion [10] and efforts to build a research culture in General Practice [14]. Despite these suggested solutions, many projects have great difficulty with recruitment.

This paper describes a recruitment strategy where the investigators were able to recruit a significant number of subjects from general practices for a randomized controlled clinical trial (RCT) with very specific recruitment requirements and tight timelines. Reasons for taking part in the study and participants experience of the recruitment process were explored as part of a qualitative interview with a sub-set of participants.

## Methods

A randomized controlled trial of a chronic disease self-management support (CDSMS) intervention for older adults with multiple chronic diseases (ANZCTRN12609000726257) was performed between 2009 and 2011 [13]. The Human Research Ethics Committee at Flinders University approved this study (ID EC00188). In order to achieve appropriate statistical power the recruitment of 254 subjects through primary care who were over the age of 60 and had at least 2 major chronic diseases was required [14]. The recruitment for this study had to be completed in seven months due to time limitations imposed as a condition for accepting funding for the study. The intervention involved 3 home visits and 4 telephone calls; pre- and post- study assessments were conducted by separate study personnel. Participation of multiple general practices was required to obtain sufficient numbers of subjects and to enhance generalizability. Once practices were recruited, participants were then recruited within each practice and provided with either a CDSMS intervention or an attention control intervention that included the provision of disease specific educational materials. In order to encourage recruitment of practices and patients, several strategies were adopted in the study protocol and are discussed below.

### Designing the study to minimize the impact of the research on day-to-day operations of the general practice

To avoid placing additional burden on general practice workloads, the study was designed to have minimal impact on the day-to-day operations of clinics. General practitioners and clinical staff were not required to change their current patient practices as additional clinical staff were employed as part of the research project to perform the CDSMS or attention-control group interventions. However general practice staff were informed that some patients might seek additional input into their care as a result of the intervention.

Clinic staff were assisted in identifying potential participants by study research staff through the use of the Pen Computer Systems Clinical Audit Tool® (CAT) [15]. All of the general practices contacted had a copy of the Audit tool previously provided by the local Division of General Practice to assist with quality improvement. However most had only occasionally used the software so they were uncertain as to how to use the software for recruitment purposes. Research staff from the project assisted practice staff in querying the electronic databases at a time convenient to the practice (including out of regular working hours if required, as searching of the database could slow some computer networks during peak working hours).

General practitioners were given the opportunity to review eligible patient lists before potential participants were contacted. Whilst a generic participant information letter was used in the study, general practitioners were given the opportunity to modify the information contained within the letter. As the process of mail merging letters is both time consuming and requires some technical skills, research staff assisted the practices with generating personalised letters on practice letterhead on site. This facilitated rapid generation of the letter of invitation and minimized the burden on general practices.

### Engaging general practices in the research

When general practice participation was sought for the study, the investigators identified practices that had an affiliation with Flinders University (the academic program conducting the research project) primarily through their mentorship of medical students or prior participation in research. Through these professional connections general practitioners and/or practice managers were identified who were responsible for decision-making regarding involvement in research. A Principal Investigator and study coordinator made brief presentations to potential practices regarding the value of this study at a time convenient for the practice. These sessions included both general practitioners and practice staff and were generally held at midday; food was provided to encourage attendance.

At these sessions the presenters highlighted that the study was a legitimate scholarly exercise with important clinical implications as the federal government was committed to promoting and funding self-management support programs in primary health care settings. Presenters emphasized that despite government enthusiasm for this type of program the evidence base was limited, particularly in older people with multiple chronic conditions who require the majority of chronic disease care. It was suggested that the participation of practices and patients would significantly contribute to the evidence regarding the effectiveness of these interventions. A one-page summary of the study and expectations for the practice was also provided.

General practitioners indicated that older patients with multimorbidity formed a large portion of their practice increasing the relevance of the study to their own work. During the presentations it was highlighted that general practitioners or practice staff were not required to directly deliver the interventions. It was also noted that all participants would receive an intervention (CDSMS or Attention Control group) and that they would all have contact in their homes with an interested health professional. While early discussions with general practitioners indicated a substantial skepticism regarding CDSMS, most felt that their patients would benefit from additional attention. The fact that both groups would receive the attention of an experienced healthcare professional eased concern of the practice staff that patients might feel that they were disadvantaged by being in the control group and therefore potentially ask the general practitioner for additional support. Finally, reimbursement was provided for general practitioners and practice staff for their time assisting with identification of potential subjects at rates used by the local general practice organization (GP Division).

### Making the research attractive to participants

To make the research attractive to participants, the letter of invitation to participate in the study used the approach advocated by the Dillman [16] to enhance response for surveys. Patients received a signed letter from their general practitioner endorsing the study but also indicating that participation was entirely voluntary. An appeal to the patient’s altruism - stressing the important contribution older patients could make to improve knowledge of how to best manage chronic diseases - was made in the cover letter and subsequent contact with study personnel. Subjects were also provided with a prepaid card to return if they were interested in receiving further information on the project.

On receiving the reply-paid card, study personnel contacted potential participants promptly by telephone, describing the study to patients as well as confirming eligibility. As there was an active comparison group, participants were informed that they would receive one of two interventions. As the study was to be performed within the patient’s home this eliminated expenses associated with travel. The stress of transportation on older adults with multimorbidity (who frequently experience associated impaired mobility and low levels of energy) was also removed.

### Minimizing attrition from the study

Minimizing attrition is a key factor in trials to ensure sufficient numbers of subjects are available for analysis to meet a priori expectations regarding power to detect differences in study groups [17]. Research staff interacting with subjects were chosen for their previous experience of working with older adults with chronic disease and possessed excellent social skills. As both study interventions included monthly visits or telephone calls by clinical study personnel this facilitated ongoing participation. In addition to this regular contact, holiday and end of study greeting cards were sent to participants thanking them for their involvement in the project. Given the high risk of mortality in this study population, the study criteria excluded participants with a life limiting illness, thus minimizing death as a cause of non-completion.

### Having recruitment as a major focus of the management of the study

Prior to initiation of the study, recruitment was identified as a key factor for success. A full-time research staff member was placed in charge of recruitment and additional staff to assist with this process were included in the budget for the project. Rolling waves of recruitment letters were sent such that contact with potential subjects could occur soon after they returned the reply card. A monthly recruitment report was created and circulated to all relevant staff to track progress with actual versus planned participant recruitment. When study personnel met for regular meetings, recruitment was always placed as the first item for discussion.

### Qualitative analysis

Qualitative interviews were conducted with a subsample of participants to further elucidate our understanding of the impacts of the various recruitment strategies. The data presented here is a secondary analysis of data collected as part of a larger qualitative investigation of participants’ experiences of CDSMS. All participants were asked their reasons for taking part in this study and their responses were explored briefly by the interviewer. Interviews were conducted by skilled qualitative researchers who were not involved in the initial recruitment to the RCT or the delivery of the RCT intervention.

Interviews were undertaken with 30 participants who were in the CDSMS arm of the study as part of the larger evaluation of the intervention. Participants were selected for qualitative interviews using stratified purposeful sampling. They were stratified into one of 3 groups (positive outcome, no change, worse outcome) as they completed the final 6 month assessment of the trial, based on their scores on questionnaires used to assess the outcomes of the RCT. The questionnaires used to determine impact included the Partners in Health (PIH) scale [18] and participant’s perceived change in health status over the past 6 months. Participants were then selected between strata and within strata for maximal variation in terms of age, gender, marital status, depression score, number of chronic conditions, and clinician. Sampling continued until 30 interviews had been completed and no new information was found to emerge from the interviews.

Those selected were contacted by the qualitative researchers and asked if they would be willing to take part in an interview about their experiences in the trial. Of those eligible, 6 declined and 26 were not selected as they were either too similar to those already interviewed (n=15); the final RCT outcome visit was more than 3 months ago (n=5); participant was still employed (n=2) or; was too unwell or could not remember the intervention (n=3). Only 1 eligible participant could not be contacted.

Interviews were tape recorded using a digital tape recorder with the permission of the interviewee(s) then transcribed verbatim. Participant’s responses were analysed using thematic analysis and were coded utilizing NVivo software. The initial analysis was completed by CB and reviewed by RR. Emerging interpretations were discussed and contested by a third author (LR) until agreement was reached. The final coding and selection of participant quotes was verified by another qualitative researcher involved with the larger study but not the current analysis (SM).

## Results

Five out of the six (83.3%) general practices contacted agreed to take part in the study. Letters of invitation were subsequently sent to 1663 potentially eligible patients and 634 (38%) responded to this letter expressing a desire for further information about the study. Following assessment just under half of these patients were excluded due not meeting the eligibility criteria. Only a very small number of patients (n = 18) who were eligible to participate declined to do so after learning more about the study. Ninety-three patients who responded were not assessed because the sample size had been reached and were sent a letter thanking them for their interest in the study.

**Figure 1:**
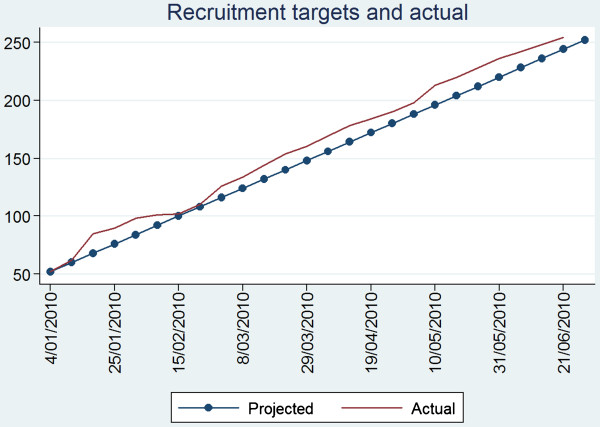
Recruitment of participants expected versus actual numbers recruited.

Despite the high complexity of recruitment, recruitment goals were met slightly ahead of time as noted in Figure 1. Attrition from both study arms were small, with only 4.8% of participants dropping out in the intervention group and 5.8% in the attention control period predominantly due to worsening health.

### Qualitative findings

Twenty-eight participants described reasons for their participation in the study. Qualitative interviews with participants revealed three primary reasons for participation. Similar proportions of participants reported participating in the study for: 1) altruistic reasons, 2) in the hope of health gains and 3) on the recommendation of their doctor. Participant quotes illustrating these themes are presented in Table 1.

**Table 1:** Quotes from qualitative interviews illustrating themes

**Theme**	**Example of comment**
1 Altruism	“I said, ‘wow, better than nothing’. People care, and if it gives us some positives at the end at some stage, and gives maybe someone in the government a bit of a rocket about the realities of life, then I reckon it’s all worthwhile.” **ID 59, male, 60yrs**
	“I thought, well, if I can help someone else, great. I’d not wish it (participant’s health problems) on anyone.” **ID 25, male, 73yrs**
	“like (my wife) said, hey, if it helps society…the same as giving blood, that sort of thing, it helps.” **ID 31, male, 69yrs**
2. This study will help me	Interviewer: ‘What made you say, yes, that’s ok, rather than no, I’m too busy?”
2.1 Expectation of health gain	Participant: ‘Well, I thought I might get some benefit out of it, you go in for something, you don’t really know what you will get out of it?” **ID 17, male, 77yrs**
2.2 Hope for future benefit for self	“I was under the impression that the purpose was to find out what can be done to help people in this situation. I thought now, ok, we don’t desperately need this help at the moment, but in the future we might…” **ID 26, male, 79yrs**
2.3 Psychosocial support	Participant: “I got a letter from my GP and I decided to participate”
	Interviewer: “What was your motivation for participating?” Participant: “I thought I need to do something!” Interviewer: “For yourself, or others?”
	Participant: “For me!” **ID 539, female, 64yrs**
	Participant: “I thought it would be a good idea. I had a letter from my GP about it, and I thought it would be a good idea to follow it…Because at that particular time I had been having a lot of health problems. My son had had a lot of health problems, and life was pretty tough. I felt that I could use some help.
	Interviewer: Did you think it might be of some additional benefit to you? Participant: “Yes, I did, because I felt pretty isolated and vulnerable at the time…and trying to get any assistance was pretty tough going.”
	**ID 782, female, 76yrs**
2.4 To gain knowledge	“I thought about it, and I thought ‘I’m going to have a talk with my GP and see exactly who’s dobbed me in.’ I had no objections, so I thought I might gain some insight into (my health problem). So I thought I’d go ahead.” **ID 558, female, 69yrs**
3. Doctor recommended study	Interviewer: do you remember why you decided to take part in the study?Participant: “Probably because of the very fact that my GP asked me to participate. He has asked me in the past to participate in other studies as well, which I’ve always agreed to. …I owe him a great debt, so I figure anything he asks me to do, I’m more than happy to participate.” **ID 59, male, 60yrs**
	“the first thing was the letter from the local doctor (which) said that he had been approached for recommendations of patients who could possibly assist in the research. He suggested that I might consider it, I can recall. I had no reason not to, so I rang the (university researchers) to tell them that I would take part.” **ID 26, male, 79yrs**

Participants who indicated they took part for altruistic reasons sought no immediate personal gain, but were motivated that the research findings could help others experiencing chronic disease.

Participants who indicated they took part with the anticipation that the study might have benefits to their well-being or future health care hoped that the study would help them either with a specific problem or generically, that it could lead to improved health care for themselves, that participation would provide additional psychosocial support, or that it would help them gain knowledge about their condition.

Finally participants who reported that they took part in the study on the recommendation of their general practitioner described how the relationship with, and the respect they held for their general practitioner was an important element of their decision to take part in the research.

## Discussion

Recruitment was successful in this study through systematically addressing a range of barriers identified in the literature. We were able to recruit the required number of participants from five general practices within a seven month period.

The study was designed to minimize to the greatest extent possible the burden on staff as this has been identified in the literature as a major barrier to participation [19]. An important and relevant research topic was chosen for which there were substantial numbers of potentially eligible patients in each practice. As Australian general practices are compensated through a fee-for-service payment methodology,any time spent on research was time not earning revenue. Hence, compensation for this time was identified as a key factor in facilitating participation.

The study was also designed to enhance recruitment by choosing an active control group approach. One factor identified in the literature is concern by general practitioners regarding randomisation, as their preference is to allocate patients based on their personal choices and customary practice [5] particularly if one of the treatments does not involve drugs [2]. Furthermore, prior research indicates uncertainty among general practitioners regarding the best way to promote CDSMS to patients [20]. In this project the interventions did not appear to have a clear advantage of one treatment over another, and were therefore perceived as being potentially beneficial

To make the research attractive to potential participants, elements of the Dillman methodology [15] were used in the mailed letters of invitation. Literature on recruitment confirms the widely held belief that general practitioners are important sources of information and that a personal expression of interest and request for participation would be more powerful than a generic letter. Altruism is a potent motivator for participation in research projects [21,22]. The important contribution older patients could make to improve our knowledge of multimorbidity was therefore emphasized [23]. This factor was further supported by the subsequent qualitative interviews showing that altruism was identified by participants as a key factor for engagement in the study.

It is interesting to note that despite care to not emphasize potential personal outcomes of the intervention during the recruitment process; this was a common factor reported by participants in the decision to take part in the study. It is reassuring, however, to note that study participants in both the treatment groups subsequently felt that they had benefited from either intervention (data not presented). Furthermore, in order to minimize attrition, research and clinical staff that had direct interaction with participants were chosen for their previous experience and excellent social skills and the study design included regular contact with participants in both intervention and attention control groups. Successful engagement with participants during the intervention period facilitated a very high rate of retention of subjects for follow-up measures in both groups [24]. Thus the design of the project facilitated both retention as well as recruitment.

### Limitations of the study

As with any recruitment strategy, where a series of decisions have to be made, there is a need for balancing ease of recruitment with other relevant study design issues. By endeavoring to minimize expectations for general practice staff and employing clinical staff to undertake the interventions, this may have also limited the potential impact on participants, as the CDSMS was not integrated into usual practice. Initiatives in this area have suggested that integration of community-based CDSMS programs into mainstream health care is unlikely to be achieved without greater general practitioner involvement during their implementation [25]. However, recruitment to these projects has previously been challenging [3,26,27] and interviews with general practitioners indicate limited understanding of CDSMS [28] and relatively low commitment to its implementation [25,29]. The model proposed in this study mirrors current practice in South Australia where CDSMS is provided upon referral but neither connects to specialist or primary care services. For studies that require GPs or practice staff to more substantially change how they provide care to their patients, the strategy of minimizing the impact of this intervention to the greatest extent possible is still applicable but needs to be balanced by the need to insure that the intervention is implemented to the fullest extent possible.

It is important to acknowledge that the results presented in this study were generated from a small number of General Practices within a relatively confined geographical area of metropolitan Adelaide. Importantly they were also General Practices where there was a pre-existing productive working relationship to members of the research team. Clearly caution is required in generalising from the present sample to General Practices in other health regions, particularly those outside Australia. In this respect, we envisage that the five strategies outlined in this paper cannot be implemented in a “cookbook fashion” and will require tailoring and adaptation to best fit local circumstances. We believe however that attention to the general concepts underlying the strategies will be widely applicable and of benefit to improving the recruitment process from general practices in many countries.

## Conclusion

In summary, the success of recruitment in this study is attributed to a research design sensitive to the realities of patient recruitment issues. This approach was paired with a research team with strong technical and interpersonal skills that facilitated work with general practices, their electronic patient databases and the participants enrolled from these practices. The success in recruiting practices built on good existing working relationships with general practices in the region. Recruitment for future studies based in General Practice might benefit from some of the approaches used for recruitment in this study.

## Competing interests

The authors declare that they have no competing interests.

## Authors’ contributions

RR drafted the manuscript. RR, LR and JO were involved in the conception and design of the study. CB: Collected and analyzed the qualitative data. LI assisted with the literature review. All authors provided valuable intellectual contributions to the manuscript during revisions. Finally, all authors have read and approved the final version of the manuscript for publication.

## Pre-publication history

The pre-publication history for this paper can be accessed here:

http://www.biomedcentral.com/1471-2296/14/125/prepub

## References

[B1] Askew DA Schluter PJ Gunn JM Research productivity in Australian general practice: what has changed since the 1990s? Med J Aust 2008 189 2 103 104 18637781 10.5694/j.1326-5377.2008.tb01931.x

[B2] Hunt CJ Shepherd LM Andrews G Do doctors know best? Comments on a failed trial Med J Australia 2001 174 3 144 146 11247619 10.5694/j.1326-5377.2001.tb143189.x

[B3] Bower P Wilson S Mathers N Short report: How often do UK primary care trials face recruitment delays? Fam Pract 2007 24 6 601 603 17872907 10.1093/fampra/cmm051

[B4] Gray RW Woodward NJ Carter YH Barriers to the development of collaborative research in general practice: a qualitative study Br J Gen Pract 2001 51 464 221 222 11255904 PMC1313954

[B5] Fairhurst K Dowrick C Problems with recruitment in a randomized controlled trial of counselling in general practice: causes and implications J Health Serv Res Policy 1996 1 2 77 80 10180853 10.1177/135581969600100205

[B6] Ross S Grant A Counsell C Gillespie W Russell I Prescott R Barriers to participation in randomised controlled trials: A systematic review J Clin Epidemiol 1999 52 12 1143 1156 10580777 10.1016/s0895-4356(99)00141-9

[B7] Salmon P Peters S Rogers A Gask L Clifford R Iredale W Dowrick C Morriss R Peering through the barriers in GPs' explanations for declining to participate in research: the role of professional autonomy and the economy of time Fam Pract 2007 24 3 269 275 17504773 10.1093/fampra/cmm015

[B8] Tognoni G Alli C Avanzini F Bettelli G Colombo F Corso R Marchioli R Zussino A Randomised clinical trials in general practice: lessons from a failure BMJ 1991 303 6808 969 971 1954424 10.1136/bmj.303.6808.969 PMC1671349

[B9] Dormandy E Kavalier F Logan J Harris H Ishmael N Marteau TM Maximising recruitment and retention of general practices in clinical trials: a case study Br J Gen Pract 2008 58 556 759 766 i-ii 19000399 10.3399/bjgp08X319666 PMC2573972

[B10] Ngune I Moyez J Dadich Sriram D Lotriet J Effective recruitment strategies in primary care research: a systematic review Quality in Primary Care 2012 20 115 23 22824564

[B11] Ellis SD Bertoni AG Bonds DE Clinch CR Balasubramanyam A Blackwell C Chen H Lischke M Goff DC Jr Value of recruitment strategies used in a primary care practice-based trial Contemp Clin Trials 2007 28 3 258 267 17030154 10.1016/j.cct.2006.08.009 PMC3760001

[B12] Bell-Syer SE Moffett JAK Recruiting patients to randomized trials in primary care: principles and case study Fam Prac 2000 17 2 187 191 10.1093/fampra/17.2.187 10758084

[B13] Bower P Wallace P Ward E Graffy J Miller J Delaney B Kinmonth AL Improving recruitment to health research in primary care Fam Pract 2009 26 5 391 397 19549623 10.1093/fampra/cmp037

[B14] Reed RL Battersby M Osborne RH Bond MJ Howard SL Roeger L Protocol for a randomised controlled trial of chronic disease self- management support for older Australians with multiple chronic diseases Contemp Clin Trials 2011 32 6 946 952 21864719 10.1016/j.cct.2011.08.001

[B15] PEN Clinical Audit Tool PEN Computer Systems 2012 http://www.clinicalaudit.com.au/ (accessed 8 Aug 2013)

[B16] Dillman DA Smyth JD Christian LM Internet, Mail, and Mixed-Mode Surveys: The Tailored Design Method 2009 3 Hoboken New Jersy: John Wiley & Sons, Inc

[B17] Robinson KA Dennison CR Wayman DM Pronovost PJ Needham Systematic review identifies number of strategies important for retaining study participants Journal of Clinical Epidemiology 2007 60 757 765 17606170 10.1016/j.jclinepi.2006.11.023 PMC1997303

[B18] Battersby MW Ask A Reece MM Markwick MJ Collins JP The Partners in Health scale: the development and psychometric properties of a generic assessment scale for chronic condition self-management Aust J Prim Health 2003 9 41 52

[B19] Halbert JA Silagy CA Finucane P Withers RT Hamdorf PA Recruitment of older adults for a randomized, controlled trial of exercise advice in a general practice setting J Am Geriatr Soc 1999 47 4 477 481 10203125 10.1111/j.1532-5415.1999.tb07242.x

[B20] Blakeman T Macdonald W Bower P Gately C Chew-Graham C A qualitative study of GPs' attitudes to self-management of chronic disease Br J Gen Pract 2006 56 527 407 414 16762121 PMC1839014

[B21] Mattson ME Curb JD McArdle R Participation in a clinical trial: the patients' point of view Control Clin Trials 1985 6 2 156 167 4006489 10.1016/0197-2456(85)90121-7

[B22] Lovato LC Hill K Hertert S Hunninghake DB Probstfield JL Recruitment for controlled clinical trials: literature summary and annotated bibliography Control Clin Trials 1997 18 4 328 352 9257072 10.1016/s0197-2456(96)00236-x

[B23] Barton CA May C Mészáros D Matheson MC Jenkins M Giles G Hopper J Walters EH Dharmage SC Abramson MJ Reasons for ongoing participation in a longitudinal cohort study Aust NZ J Public Health 2012 36 4 397 398

[B24] Lawton J Fox A Fox C Kinmonth AL Participating in the United Kingdom Prospective Diabetes Study (UKPDS): a qualitative study of patients' experiences Br J Gen Pract 2003 53 490 394 398 12830569 PMC1314601

[B25] Kennedy A Gately C Rogers A EPP Evaluation Team: Expert Patients Programme: Assessing the Process of Embedding EPP in the NHS: Preliminary Survey of PCT Pilot Sites (National Evaluation) 2004 Manchester: National Primary Care Research and Development Centre 1 46

[B26] Blackberry ID Furler JS Young D Best JD What does it cost to establish a practice-nurses-led clinical trial in general practice? Med J Aust 2009 191 9 492 495 19883343 10.5694/j.1326-5377.2009.tb02911.x

[B27] Horsburgh MP Bycroft JJ Mahony FM Roy DE Miller DJ Goodyear-Smith FA Donnell EC The feasibility of assessing the Flinders Program of patient self-management in New Zealand primary care settings J Prim Health Care 2010 2 4 294 302 21125070

[B28] Rogers A Kennedy A Nelson E Robinson A Uncovering the limits of patient-centeredness: implementing a self-management trial for chronic illness Qual Health Res 2005 15 2 224 239 15611205 10.1177/1049732304272048

[B29] Harris MF Williams AM Dennis SM Zwar NA Powell Davies G Chronic disease self-management: implementation with and within Australian general practice Med J Aust 2008 189 10 Suppl S17 20 19143580 10.5694/j.1326-5377.2008.tb02204.x

